# Comparing the Metabolic, Systemic, and Neuropsychiatric Impacts of Olanzapine and Clozapine in Patients with Schizophrenia

**DOI:** 10.3390/ph18091314

**Published:** 2025-09-01

**Authors:** Nayef Samah Alharbi, Noha Alaa Hamdy, Esam M. Aboubakr, Mansour Alharbi, Mostafa A. Ali, Ghaleb Alharbi, Ahmed Ibrahim ElMallah

**Affiliations:** 1 Eradah and Mental Health Hospital, Ministry of Health, Buraidah 52386, Saudi Arabia; gs-nayef.alharbi@alexu.edu.eg; 2 Department of Clinical Pharmacy and Pharmacy Practice, Faculty of Pharmacy, Alexandria University, Alexandria 21521, Egypt; noha.alaaeldine@alexu.edu.eg; 3 Department of Pharmacology and Toxicology, Faculty of Pharmacy, South Valley University, Qena 83523, Egypt; 4 Department of Psychiatry, College of Medicine, Qassim University, Buraidah 52571, Saudi Arabia; m_alharbi@qu.edu.sa; 5 Department of Psychiatry, College of Medicine, Assuit University, Assiut 71515, Egypt; mostafazim@med.aun.edu.eg; 6 Department of Clinical Pharmacy, College of Pharmacy, Shaqra University, Shaqra 11961, Saudi Arabia; g.alharbi@su.edu.sa; 7 Department of Pharmacology and Toxicology, Faculty of Pharmacy, Alexandria University, Alexandria 21521, Egypt; ahmed.elmallah@alexu.edu.eg

**Keywords:** clozapine, olanzapine, PANSS, inflammation, schizophrenia

## Abstract

**Background/Objectives**: The clinical impact of antipsychotics on the human body remains inadequately investigated, hence we aimed to compere the effects olanzapine (OLZ) and Clozapine (CLZ) on different body systems. **Methods:** 48 patients and 24 healthy individuals were involved, and followed over six months. PANSS, metabolic, cardiovascular, inflammatory, and neuronal transmitter parameters were determined. **Results:** No significant difference was found between the effects of the two drugs on blood mineral and cardiovascular parameters, except for CK-MB, which showed a greater increase in the OLZ group than in the CLZ group. Both drugs increased the lipid profile and HbA1C levels, with the effect of CLZ being more prominent. Both drugs increased the patients’ body weights, with no significant difference between their effects. Regarding renal and hepatic functions, OLZ had a more notable effect on creatinine and albumin levels than CLZ, while AST and ALT showed markedly greater increases in the CLZ-treated group than in the OLZ-treated group. Regarding the effects on neurotransmitters and inflammatory mediators, both drugs increased serotonin and ghrelin levels, in addition to decreasing leptin concentrations, and decreased the inflammatory mediators IL-1β, IL-6, and –TNF-α, with the effect of OLZ being more prominent. Regarding therapeutic efficacy, CLZ was more effective at reducing general and negative symptoms than OLZ. **Conclusions:** The present study revealed that CLZ had a greater impact on metabolic parameters and better therapeutic efficacy in attenuating both general and negative symptoms, whereas OLZ had more detectable anti-inflammatory effects, aid determining the appropriate treatment for schizophrenic patients.

## 1. Introduction

Psychotic illnesses, such as schizophrenia, bipolar disorder, depression with psychotic characteristics, and substance-induced psychoses, are being increasingly acknowledged as significant global health concerns [1,2]. Schizophrenia is one of the top 20 causes of years lived with disability worldwide. This is mainly because it tends to start at a young age, has a significant impact on functioning, and often persists for a long time [3]. Individuals diagnosed with psychosis often experience a high prevalence of physical comorbidities, make unhealthy lifestyle choices, and often have various cardiovascular risk factors. These variables, together with inequities in physical healthcare and symptoms associated with the disease, contribute to the substantial burden and premature mortality of patients with psychosis [4].

Metabolic syndrome and other cardiovascular risk factors are very common in individuals diagnosed with schizophrenia. Approximately one-third of individuals with schizophrenia have metabolic syndrome, which includes obesity, hyperlipidemia, hyperglycemia, and hypertension [5]. Studies have shown that individuals with schizophrenia have a life expectancy that is about 25 years shorter than that of the general population: Suicide and other non-natural causes contribute to about 40% of this increased mortality, and approximately 60% of premature deaths are due to other causes, such as cardiovascular and metabolic diseases [6,7].

However, researchers and clinicians continue to face persistent difficulty in the pharmacological treatment of psychosis. Despite the recent increase in the availability of newer antipsychotic drugs known as atypical antipsychotics, current medications are still not ideal for properly treating this disorder. Atypical antipsychotics have a lower incidence of extrapyramidal symptoms (including involuntary movements, muscle stiffness, and tremors) and tardive dyskinesia [8,9]. However, there are increasing concerns about the considerable long-term metabolic and cardiac side effects associated with these antipsychotic medications, which have not been fully elucidated. Atypical drugs vary regarding their tendency to cause these negative side effects, and medical professionals should consider the balance between the risks and benefits of each drug for each patient [10]. It is necessary to conduct baseline examinations and regular follow-ups of patients, as well as identify those at risk of obesity, diabetes, and cardiovascular morbidity. Successful antipsychotic medication choice relies on the crucial aspect of making well-informed decisions [11,12].

Clozapine (CLZ) is considered the prototype of atypical antipsychotics, and it has been regarded as the most significant breakthrough in the treatment of schizophrenia since the identification of the initial antipsychotic medications [13].

CLZ has a low affinity for the dopamine D2 receptor, exerting an antagonistic effect and reducing positive symptoms, and it has a higher affinity for dopaminergic D4, serotonergic 5HT2A, and receptors with antagonistic action, thereby reducing negative symptoms. Furthermore, it weakly interferes with dopamine D1, D3, and D5 receptors, and it has a high affinity for alpha-adrenergic, cholinergic, histamine (H1), muscarinic, and serotonergic receptors [14]. Nevertheless, the widespread application of CLZ in clinical settings is relatively restricted due to the potential development of severe side effects, including agranulocytosis and thrombosis [15]. CLZ has been linked to fatal myocarditis and cardiomyopathy in young individuals with good physical health [16]. The clinical use of CLZ gives rise to many concerns due to clinician variables such as a limited understanding and worries regarding adverse drug effects (ADEs), which greatly contribute to hesitancy in its use as treatment; the variations in its pharmacological effects have not been fully elucidated [13].

Olanzapine (OLZ) is a commonly prescribed atypical antipsychotic licensed by the FDA for the treatment of bipolar disorder, schizophrenia, and related depression. Its affinity for 5-HT2A serotonin receptors is higher than that for dopamine D2 receptors, and it also has a high affinity for adrenergic (alpha 1), histamine (H1), and muscarinic (M1-2 and M4-5) receptors [17]. It functions as an antagonist on dopamine D2 receptors in the mesolimbic pathway, preventing dopamine from exerting its effects on the post-synaptic receptor. Moreover, it acts as an antagonist on serotonin 5HT2A receptors in the frontal cortex, and it exhibits a weak binding affinity for these receptors and readily dissociates, hence facilitating regular dopamine neurotransmission [18]. Although OLZ presents the greatest risk for developing metabolic syndrome, it exhibits fewer extrapyramidal symptoms than other first- and second-generation antipsychotics [19].

Regarding the differences between CLZ and OLZ in terms of their side effects, CLZ appears to elicit a lower increase in prolactin levels than olanzapine, and it is linked to a greater occurrence of leukopenia (decreased white blood cell count), excessive salivation, drowsiness, and seizures [20]. No sufficient studies have compared the differences in the inflammatory and metabolic effects of clozapine and olanzapine in terms of weight gain, blood glucose levels, and other metabolic side effects. Nevertheless, the credibility and relevance of related studies are limited [21].

Therefore, the present study was conducted to compare the antischizophrenic effects of CLZ and OLZ (on positive, negative, and general symptoms) in patients, with time intervals of 0, 3, and 6 months, in addition to comparing their effects on blood glucose levels, blood minerals, kidney function, liver function, neurotransmitters (serotonin, dopamine, ghrelin, and leptin), and inflammatory mediators (TNFα, IL-6, and IL-1β). In view of the obtained results, we aim to aid psychiatrists in selecting the most suitable treatment for patients with schizophrenia, especially in the presence of comorbidities that can be affected by CLZ or OLZ administration.

## 2. Results

**Figure 1:**
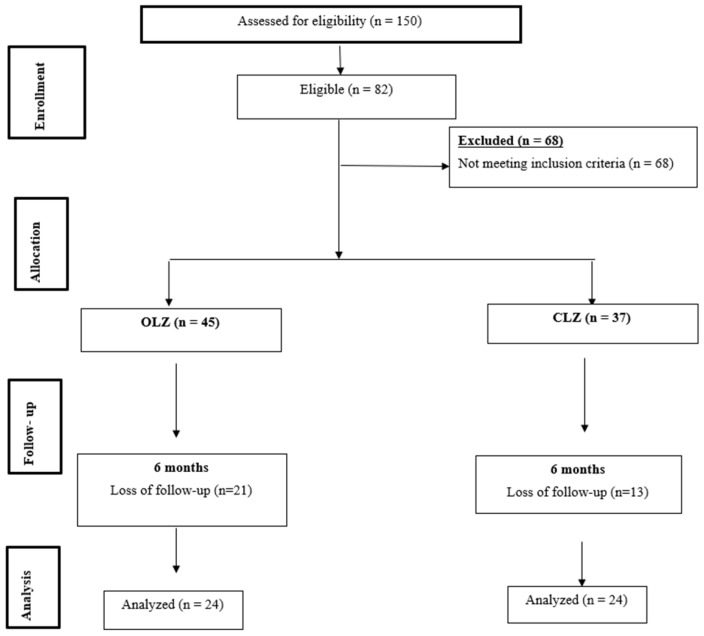
Study flowchart.

A total of 150 patients with schizophrenia admitted to the Qassim Mental Health Hospital in Saudi Arabia in the period from July 2023 to April 2024 were screened. We found that 82 patients met the eligibility criteria, of whom 45 received OLZ and 37 received CLZ. In the OLZ and CLZ groups, 21 and 13 patients were lost to follow-up, respectively (Figure 1).

### 2.1. Demographic Characteristics

The participants’ demographic characteristics were as follows: In the control group, 50% were male and 50% were female, 75% were married and 25% were not married, 70.8% were employed and 29.2% were not employed, and the mean age was 45.6 years. In the olanzapine-treated group, 54.1% were male and 45.8% were females, 79.1% were married and 20.8% were not married, 75% were employed and 25% were not employed, and the mean age was 41.8. In the clozapine-treated group, 54.1% were male and 45.8% were female, 75% were married and 25% were not married, 83.3% were employed and 16.6% were not employed, and the mean age was 44.2 years (Table 1).

### 2.2. Baseline Characteristics

Differences in the baseline characteristics of the OLZ and CLZ groups were examined. No statistically significant differences were observed between the two groups regarding the laboratory data, including the electrolyte levels, cardiac parameters, glycated hemoglobin (HbA1c), lipid profile (including total cholesterol, HDL, and LDL), kidney function, liver function, weight, and waist circumference.

The PANSS scores at baseline were statistically significantly higher in the CLZ group, mostly on the general and negative scales and to a lesser extent on the positive scale. Moreover, the cumulative score was significantly higher in the CLZ-treated group than in the OLZ-treated group; however, this did not affect its overall reduction, as the CLZ group showed a significantly higher reduction in this score after 6 months of treatment. 

### 2.3. Effects on PANSS Score

#### 2.3.1. Effects on General Symptoms

After 3 months of treatment, there were no statistically significant differences between the OLZ and CLZ groups (*p* = 0.098) in terms of the effect on general symptoms. Moreover, after 6 months of treatment, we did not find any statistically significant difference between the OLZ and CLZ groups in terms of the general scores (*p* = 0.124) (Table 2).

Table 3 shows that when comparing the effects of the oral administration of CLZ and OLZ for 6 months based on the percentage changes in the total scores, we found that the former caused a 52% reduction in these scores, whereas the latter caused a 31% reduction (*p* = 0.187); however, this difference was not statistically significant.

#### 2.3.2. Effects on Negative Symptoms

Regarding the effects on negative symptoms, a statistically significant difference was observed between the OLZ and CLZ groups after 3 months of treatment (*p* = 0.039) but not after 6 months of treatment (*p* = 0.11) (Table 4).

Table 5 shows that when comparing the effects of the oral administration of CLZ and OLZ for 6 months based on the percentage changes in the total scores, we found that the former had a more prominent effect on negative symptoms than the latter. That is, we found a 35% reduction in the total score of the CLZ group and a 22% reduction in that of the OLZ group (*p* = 0.11); however, this was not statistically significantly different.

#### 2.3.3. Effects on Positive Symptoms

After 3 months of treatment, there was no statistically significant difference (*p* = 0.325) between the OLZ and CLZ groups. Moreover, after 6 months of treatment, there was no statistically significant difference between the OLZ and CLZ regarding positive symptoms (*p* = 0.542) (Table 6 and Table 7).

Table 7 shows that when comparing the effects of CLZ and OLZ after 6 months of treatment, we found that the percentage reductions in the total scores were not significantly different between the CLZ and OLZ groups (*p* = 0.493).

#### 2.3.4. Effect on Total PANSS Score

As shown in Table 8, the total PANSS score of the OLZ-treated patients decreased from 85.35 to 57.8 after 3 months and to 48.9 after 6 months, while that of the CLZ-treated group decreased from 109.64 to 77.46 after 3 months and to 50.66 after 6 months.

### 2.4. Effect on Blood Minerals

Table 9 shows that when comparing the effects of clozapine and olanzapine on blood mineral concentrations at the 6-month follow-up, we found a non-significant difference between the results of the two drugs; however, the increase in potassium levels was more notable in the CLZ group than in the OLZ group.

### 2.5. Effect on the Metabolic Parameters

Table 9 and Table 10 show that by comparing the effects of CLZ and OLZ after 6 months of administration, we found a significant increase in the HbA1C (15%) and LDL (16%) levels in the CLZ-treated group compared with those in the OLZ-treated group (5% and 4%, respectively).

### 2.6. Effect on Renal and Hepatic Functions

When comparing the effects of OLZ and CLZ on both hepatic and renal functions, which included urea, creatinine, total bilirubin, direct bilirubin, ALT, AST, alkaline phosphatase, and albumin, we found a significant difference in the obtained results for the kidney function test, where OLZ had a significant increasing effect on creatinine levels compared with CLZ (*p* = 0.032), while in the hepatic function tests, CLZ had a significant increasing effect on ALT and total bilirubin compared with OLZ (*p* = 0.038, 0.044, and 0.041, respectively), while OLZ had a significant decreasing effect on albumin compared with CLZ (*p* = 0.03) (Table 9 and Table 10).

### 2.7. Effect on Cardiovascular Parameters

Table 11 and Table 12 show that the oral administration of OLZ and CLZ for 6 months did not have a significant effect on ECG (QTc), systolic blood pressure (SBP), diastolic blood pressure (DBP), or heart rate (HR), and both drugs had similar effects. However, when we examined CK-MB after 6 months of OLZ and CLZ administration, we found a non-statistically significant increase in the concentration of this enzyme in both groups of patients. Moreover, although the effect was more prominent in the OLZ-treated group, it did not reach statistical significance (*p* = 0.325) when compared with that in the CLZ-treated group.

### 2.8. Effects on Body Weight and Waist Circumference

We found significant increases in body weight and waist circumference in both the OLZ and CLZ groups after 6 months of treatment compared with baseline (Table 13 and Table 14).

### 2.9. Effects on Neurotransmitters

#### 2.9.1. Effects on Serotonin

**Figure 2:**
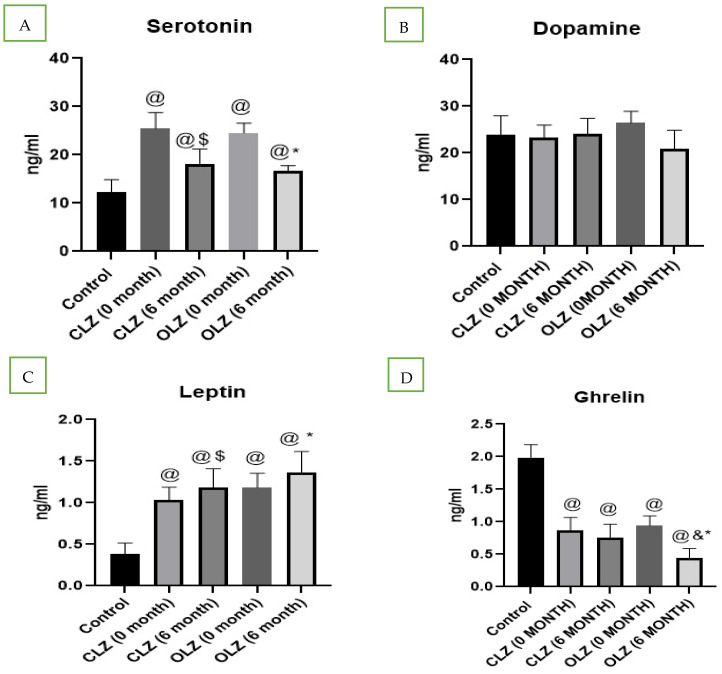
Analysis of biochemical parameters in controls, patients with schizophrenia, and patients with schizophrenia after treatment: (**A**) serotonin, (**B**) dopamine, (**C**) leptin, (**D**) ghrelin. @—significantly different in comparison to the control group, $—significantly different in comparison to the CLZ group (0 months), & significantly different in comparison to the CLZ group (6 months), *—significantly different in comparison to the OLZ group (0 months). Results are presented as the mean ± SEM (n = 24).

As shown in Figure 2A, the serum serotonin levels of healthy controls and patients with schizophrenia were examined. We found that, before starting treatment with CLZ (25.2 ng/mL) or OLZ (24.6 ng/mL), the serotonin levels in the patients with psychosis were significantly increased compared with those in the healthy controls (11.7 ng/mL). However, after 6 months of CLZ (18.8 ng/mL) or OLZ (18.1 ng/mL) treatment, a significant reduction (*p* < 0.05) in serotonin was observed in both groups, and the percentage reductions were similar between the two groups.

#### 2.9.2. Effects on Dopamine Levels

In the present study, we did not find any significant differences in dopamine levels in patients with psychosis compared with in controls. Moreover, no significant changes were observed in dopamine levels after 6 months of treatment with CLZ or OLZ compared with baseline (Figure 2B).

#### 2.9.3. Effects on Leptin Levels

As shown in Figure 2C, serum leptin levels were found to be higher in patients with psychosis than in controls. Furthermore, we found a significant increase in leptin levels after 6 months of treatment with both CLZ (*p* = 0.039) and OLZ (*p* = 0.044), with this effect being more prominent in the OLZ-treated group than in the CLZ-treated group.

#### 2.9.4. Effects on Ghrelin

In the present study, serum ghrelin levels were determined, and they were found to be significantly lower in patients with psychosis than in control individuals. Furthermore, we found that the ghrelin serum levels in patients treated with the oral administration of CLZ for 6 months were not significantly affected, but those in patients treated with the oral administration of OLZ for 6 months significantly decreased compared with those in patients with psychosis who were not treated, as shown in Figure 2D.

### 2.10. Effects on Inflammatory Mediators

**Figure 3:**
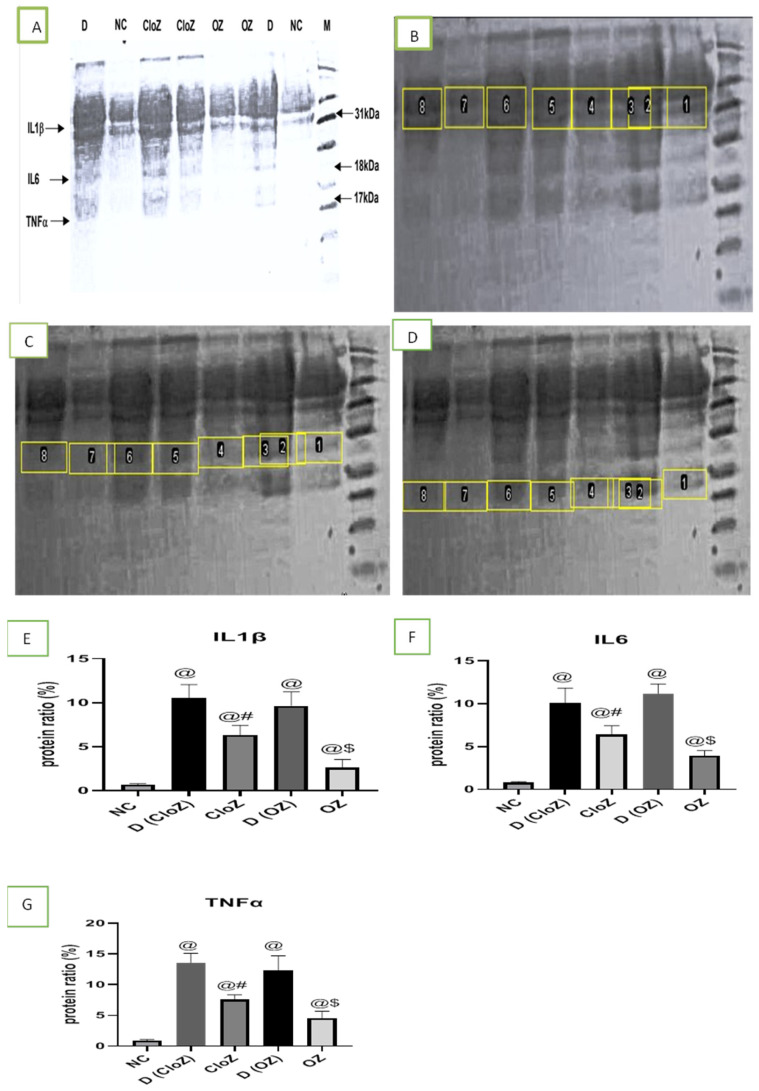
Analysis of the inflammatory mediator concentrations in controls, patients with schizophrenia, and patients with schizophrenia after treatment. (**A**) A whole Western blot image, (**B**) IL-1β Western blot image, (**C**) IL-6 Western blot image, (**D**) TNF-α Western blot image, (**E**) quantitative determination of IL-1β expression, (**F**) quantitative determination of IL-6 expression, (**G**) quantitative determination of TNF-α expression. 1 and 7 = NC = normal control; 8 = D = D (CloZ) = patients with schizophrenia before clozapine treatment; 5 and 6 = CloZ = patients with schizophrenia after 6 months of clozapine treatment; 2 = D = D (OZ) = patients with schizophrenia before olanzapine treatment; 3 and 4 = OZ = patients with schizophrenia after 6 months of olanzapine treatment. @—significantly different in comparison to the NC group at *p* < 0.05, #—significantly different in comparison to the D (CloZ) group at *p* < 0.05, $—significantly different in comparison to the D (OZ) group at *p* < 0.05. Results are presented as the mean ± SEM (n = 12).

As shown in Figure 3, by using the Western blot technique, we found notably high concentrations of the inflammatory mediators IL1β, IL-6, and TNF-α in the serum of patients with schizophrenia compared with in the controls. However, these mediators were significantly downregulated by the oral administration of both OLZ and CLZ, with this effect being more prominent in the OLZ-treated group than in the CLZ-treated group (*p* = 0.017).

## 3. Discussion

The chronic nature of schizophrenia has significant physical, social, and economic consequences, which have not been fully recognized in terms of their influence on public health [22]. It is a debilitating disease that causes the loss of productivity among those affected by it and necessitates ongoing expenses for hospitalization, therapy, and rehabilitation [22]. This chronic illness is distinguished by its initiation in early adulthood, lifelong progression, incapacitating symptoms, decline in functional capacity, and lack of social acceptance, rendering it one of the most devastating and economically exhausting diseases [23].

The chronic management of schizophrenia necessitates the prolonged use of antipsychotic medications. Inadequate effectiveness, low patient adherence, extrapyramidal symptoms, weight increase, and sedation can disrupt sustained adherence to maintenance medication treatment [24]. While atypical antipsychotic medications have been linked to a reduced likelihood of extrapyramidal symptoms, such as parkinsonism, dystonia, akathisia, and tardive dyskinesia, they also carry an increased risk of the occurrence of metabolic side effects [25]. Moreover, the potential therapeutic applications of antipsychotic medications have not been thoroughly investigated, and their clinically important side effects that could affect different body systems have not been fully explored; these are challenging tasks that require further investigation [26].

The introduction of chlorpromazine significantly improved the pharmacological management of individuals diagnosed with schizophrenia. Subsequently, further antipsychotic medications were introduced until the emergence of second-generation antipsychotics (SGAs) [26]. These novel medications, sometimes referred to as atypical antipsychotics, are believed to be more effective in managing negative symptoms and demonstrate fewer adverse effects than first-generation antipsychotics. These drugs have different pharmacological profiles, and their side effects may differ from one agent to another [27]. In the present study, we compared two of the most commonly used antischizophrenic drugs—clozapine and olanzapine—regarding their different cardiovascular, metabolic, and inflammatory effects, in addition to their impact on different PANNS score classes, which are commonly used to determine the efficacy of antipsychotic drugs.

The dopamine hypothesis postulates that schizophrenia is characterized by dopamine abnormalities in the mesolimbic and prefrontal brain areas [28]. However, subsequent studies have indicated that changes in glutamate, GABA, acetylcholine, and serotonin are also involved in the development of schizophrenia [29,30]. Nevertheless, the correlation between symptoms of schizophrenia and excessive dopamine transmission has already been debated [31,32]. The positive symptoms, which include hallucinations and delusions, are thought to result from an increased subcortical release of dopamine, which leads to D2 receptor activation and disturbances in cortical pathways by the nucleus accumbens. The negative symptoms, including anhedonia and a lack of motivation, are thought to result from decreased D1 receptor activation in the prefrontal cortex and reduced activity of the nucleus caudatus [33,34]. Currently, there exists a discrepancy between the findings from animal models when testing for positive symptoms and the latest clinical evidence regarding dopaminergic disorders in schizophrenia [35,36]. However, the effect of the administration of antipsychotic medications on both dopamine and serotonin blood levels has not been fully investigated, and the results that have been obtained are contradictory [35].

In our study, we examined the serotonin and dopamine levels in both healthy controls and patients with schizophrenia. We found that serotonin levels were significantly higher in patients with schizophrenia than in controls, whereas dopamine levels did not significantly differ. However, after 6 months of CLZ and OLZ administration, a significant reduction in serotonin levels was observed, contributing to a reduction in the positive symptoms of patients with schizophrenia; no significant difference was observed between CLZ and OLZ in terms of this effect. Regarding dopamine levels, OLZ administration had a notably stronger reducing effect than CLZ, but this difference was not statistically significant. Thus, we can conclude that both OLZ and CLZ mainly exert their pharmacological effects on the dopaminergic system by blocking its receptors without significantly affecting blood dopamine levels. 

Since its development in the mid-1980s, the Positive and Negative Syndrome Scale (PANSS) has been the predominant tool for assessing the intensity of schizophrenia and other psychotic diseases [37]. Out of the 30 items in the PANSS, seven are included in the positive scale, seven are included in the negative scale, and the remaining 16 are included in the general psychopathology scale [38].

In this study, we aimed to introduce new dimensions when comparing antipsychotic drugs that are not commonly discussed in previous studies and have clinical significance. Furthermore, related studies have stated that, when comparing antipsychotic drugs, it is crucial to analyze positive, negative, and general psychopathology subscale scores separately rather than relying solely on the total score [39,40,41]. The Positive and Negative Syndrome Scale (PANSS), widely regarded as the gold standard for assessing schizophrenia symptoms, divides symptoms into positive, negative, and general psychopathology subscales, each reflecting distinct aspects of the disorder, such as hallucinations and delusions (positive), emotional withdrawal and blunted affect (negative), and broader symptoms such as anxiety and poor attention (general). Research indicates that changes in these subscales may translate differently into clinical improvement, and total scores can obscure important variations in drug effects on specific symptom domains [41,42,43]. For instance, researchers have shown that relying on total PANSS scores may generate statistically significant but clinically moderate improvements, whereas evaluating subscale changes allows for a more nuanced and clinically meaningful interpretation of antipsychotic effectiveness [44,45]. Moreover, the prominence of negative symptoms in the PANSS can influence overall score changes, making subscale analysis essential to differentiate drug efficacy on diverse symptom clusters [44,46,47]. Thus, using subscale scores in addition to total scores enhances the clinical relevance and precision of comparisons between antipsychotic treatments.

The scores of these scales are determined by aggregating evaluations from individual component items. Accordingly, the possible range for the positive and negative scales is between 7 and 49, while that for the general psychopathology scale is between 16 and 112 [48]. CLZ exhibits effectiveness in alleviating symptoms of schizophrenia according to the PANSS. Studies have indicated significant decreases in positive symptoms (e.g., hallucinations and delusions) and improvements in negative symptoms (e.g., social retreat and dulled affect) [20], with certain studies indicating more pronounced improvements in negative symptoms relative to positive symptoms over time [20,49]. Moreover, PANSS scores of general psychopathology symptoms, which include anxiety and disorganization, demonstrate a significant decrease with the administration of clozapine, surpassing clinically significant thresholds [50]. However, OLZ has shown considerable effectiveness in alleviating both positive and negative symptoms, along with overall psychopathology, in individuals with schizophrenia, as assessed using the PANSS [51,52]. Researchers have demonstrated that olanzapine achieves superior improvements in PANSS scores compared to risperidone, especially for negative symptoms, with notable decreases evident across all scales post-treatment [53,54].

In the present study, regarding the positive scale, the percentage reduction in the total score was slightly higher in the CLZ-treated group than in the OLZ-treated group. CLZ had stronger attenuating effects on excitement, grandiosity, suspiciousness, and hostility than OLZ, whereas OLZ had stronger attenuating effects on hallucinations than CLZ. Regarding the attenuation of negative symptoms, CLZ showed significantly higher attenuation of blunted affect, emotional withdrawal, poor rapport, passive apathetic social withdrawal, difficulty in abstract thinking, and the total score, whereas OLZ was more efficient in attenuating the lack of spontaneity, the flow of conversation, and stereotyped thinking. Moreover, when comparing CLZ and OLZ, we found that CLZ had a stronger, significant mitigating effect on general symptoms, including somatic concern, anxiety, guilt feelings, tension, mannerisms and posturing, depression, motor retardation, uncooperativeness, unusual thought content, disorientation, poor attention, lack of judgment and insight, disturbance of volition, pre-occupation, active social avoidance, and total score, whereas OLZ was slightly more effective in reducing poor impulse control.

Approximately 33% of individuals diagnosed with schizophrenia also have metabolic syndrome, and the prevalence of this condition can reach up to 69% in those with chronic illness [55]. The incidence of obesity, type 2 diabetes, and hypercholesterolemia in individuals diagnosed with schizophrenia is reportedly 3–5 times higher than that in the whole population [55]. While earlier research investigated the weight and metabolic changes associated with various antipsychotic drugs, the results of the extent of these changes, specifically for CLZ and OLZ, remain uncertain and contradictory [56,57], thus requiring further investigation.

Regarding the effects of antipsychotic drugs (APDs) on blood glucose levels, experimental evidence demonstrated that APDs suppress Akt activation, leading to the development of insulin resistance in muscle cells; additionally, the effects of olanzapine on the glycogen content in L6 myotubes were demonstrated to be dependent on both dosage and time [58]. Olanzapine reduced the phosphorylation of IRS-1 induced by insulin and eliminated the changes in pPI3K, pAkt, and pGSK-3 expression induced by insulin [59]. Moreover, antipsychotic medications inhibit the brain’s capacity to detect sugar and regulate glucose metabolism in tissues [58].

The acute administration of OLZ can precipitate a rapid increase in blood glucose levels, irrespective of alterations in body weight, indicating a direct effect on glucose homeostasis [60,61]. This phenomenon is believed to encompass elevated hepatic glucose production, diminished insulin secretion, and decreased insulin sensitivity [60,62]. However, CLZ administration is associated with a heightened risk of hyperglycemia and diabetes mellitus. Different studies have shown that CLZ intake may result in irregular glucose metabolism in a significant number of patients [63,64]. The metabolic responses to OLZ and CLZ administration are heterogeneous and patient-dependent, and studies still differ on which drug poses a greater risk for each parameter [65,66]. Moreover, the precise mechanism by which clozapine influences glucose metabolism remains incompletely elucidated. It may entail the inhibition of insulin secretion, insulin resistance, or the disruption of glucose homeostasis [63,67,68].

Clozapine and olanzapine, both atypical antipsychotic drugs, exert differing influences on blood cholesterol levels [69,70]. Clozapine is linked to elevated serum triglyceride levels but does not markedly affect total cholesterol levels, as evidenced in investigations of patients with schizophrenia [63,71]. Conversely, olanzapine significantly affects lipid metabolism, resulting in dyslipidemia marked by increased triglyceride, total cholesterol (TC), and low-density lipoprotein cholesterol (LDL-C) levels in patients [70,72]. Experimental investigations indicate that olanzapine aggravates hyperlipidemia and atherosclerosis by disrupting hepatic lipid metabolism, enhancing de novo cholesterol synthesis, and hindering cholesterol clearance pathways [72].

In the present study, we found that CLZ had more notable effects on blood glucose and lipid levels than OLZ: Glycosylated hemoglobin increased by 24% after CLZ administration for 6 months, while it increased only by about 15% after OLZ administration. Additionally, cholesterol and LDL levels increased by 3% and 16% in the CLZ group, respectively, while they increased by 5% and 4% in the OLZ group, respectively.

Multiple factors contribute to the increase in body weight among individuals diagnosed with schizophrenia or psychosis. A sedentary lifestyle, poor dietary habits, genetic predisposition, and antipsychotic therapy are regarded as the primary factors contributing to this phenomenon [73]. The management of individuals treated for schizophrenia often considers the significant issue of weight gain caused by anti-schizophrenic medications. Several theories have been suggested regarding the tendency of antipsychotic medications to cause weight gain; however, the extent of the weight increase differs depending on the specific antipsychotic medication used and the unique characteristics of the patient [57,74]. In the present study, we did not observe a significant difference in the effects of CLZ and OLZ on patient body weight. We found that, after 6 months of administration, CLZ increased patient body weight by 14%, while OLZ increased it by 16%. To further explain our obtained results, the patients’ blood leptin and ghrelin hormone concentrations were measured.

Among the peptide hormones that influence metabolism, the correlation between leptin and antipsychotic drugs is not fully understood; hence, some studies have found that obesity and insulin resistance were commonly associated with increased serum leptin levels [75]. In the present study, we found a significant upregulation of the leptin hormone in patients with psychosis, and this level further increased after 6 months of CLZ and OLZ administration, with the effect of OLZ being more prominent than that of CLZ. However, the ghrelin hormone levels in patients with psychosis and the effects of antipsychotics on them have not been fully explored. Furthermore, the results of studies on the effects of OLZ and CLZ on ghrelin hormone levels are conflicting and not definitive [76,77,78]. In the present study, we found significantly lower ghrelin hormone levels in patients with schizophrenia, and they were further decreased by OLZ administration; conversely, CLZ did not have significant effects on these levels. A triphasic impact on ghrelin levels was proposed in a previous study [79]: First, ghrelin levels initially increase due to an acute upregulatory effect of CLZ and OLZ on ghrelin synthesis. Then, they decrease, which may be attributed to negative feedback resulting from the increase in body weight and food consumption caused by CLZ and OLZ. Finally, they either recover to their baseline values or increase and establish a new equilibrium.

Individuals with schizophrenia have a two-fold higher likelihood of being diagnosed with and dying from cardiovascular disease than the general population [80]. The increasing disparity in death rates between individuals with schizophrenia and the general population indicates the necessity of an improved understanding of the determinants of cardiovascular disease in this particular population [80,81]. However, the cardiovascular side effects of antipsychotic medications are highly prevalent. The effects encompass postural hypotension and tachycardia resulting from the inhibition of anticholinergic or α1-adrenoceptor mechanisms, and they can develop in most patients when administered at therapeutic doses [82]. The inhibition of calmodulin, sodium, and calcium channels and α2-adrenoceptors in the central nervous system is of debated clinical importance [83,84]. A potential unique characteristic of clozapine is its ability to cause mortality from both myocarditis and cardiomyopathy [16]. Many additional studies are needed to precisely identify high-risk medications and the specific groups that are susceptible to sudden death, together with the underlying mechanisms and the magnitude of the risk.

Electrocardiogram (ECG) is one of the most commonly used tests to evaluate cardiac muscle conduction. Regarding the use of the 12-lead ECG technique, normal QTc values are generally considered to be between 350 and 440 ms. In the present study, although both CLZ and OLZ increased the QTc values of patients with schizophrenia after 6 months of administration, they still remained at normal levels. However, we examined patients’ systolic and diastolic pressure, in addition to their heart rate, after 6 months of CLZ and OLZ administration. We found that both drugs had a non-significant effect on their blood pressure but increased their heart rate by 7% and 9%, respectively. Creatine kinase-MB (CK-MB) is an enzyme primarily found in cardiac muscle cells, and its levels increase in the case of myocardial damage. Although, in the present study, we did not find a statistically significant difference in the CK-MB levels between OLZ- and CLZ-treated patients, the elevated levels of CK-MB in OLZ-treated patients could have a significant clinical impact on cardiac patients.

The serum CK-MB concentration of patients treated with OLZ was significantly higher than that of patients treated with CLZ.

A lipid profile is a blood test commonly used by healthcare providers to monitor and screen the risk of development of cardiovascular diseases, specifically, atherosclerosis. In our study, we found a mild increase in both cholesterol and low-density lipoprotein (LDL) levels, accompanied by a significant decrease in high-density lipoprotein (HDL) levels, in patients treated with OLZ for 6 months; conversely, patients treated with CLZ showed a notably higher increase in LDL levels and significantly lower HDL levels than patients treated with OLZ.

The available data on the long-term effects of antipsychotics on blood mineral levels are somewhat limited; however, hypokalemia and hyponatremia have been identified in patients admitted to acute mental health facilities [85]. The increased incidence of hypokalemia among acutely unwell mental health patients has been hypothesized to be influenced by both agitation and the use of some antipsychotic pharmaceuticals [86]. Hyponatremia is an infrequent but significant side effect known to occur during the administration of some psychiatric medications such as aripiprazole, causing the syndrome of inappropriate antidiuretic hormone secretion (SIADH), in which water homeostasis is disrupted by a continuous secretion of antidiuretic hormone (ADH), even when there are no exogenous or endogenous triggers [85,87]. Noradrenergic, serotonergic, and dopaminergic pathways are involved in controlling the production of antidiuretic hormone (ADH), resulting in the disturbance of blood mineral levels [88]. Earlier research findings suggest that some individuals who are administered certain types of antipsychotics such as risperidone and paliperidone suffer from a reduced bone marrow density, resulting in the development of osteoporosis [89,90]. To compare the effects of OLZ and CLZ on blood mineral levels, the serum levels of sodium, potassium, calcium, chloride, and magnesium were determined. We found non-significant changes in the levels of these ions, and OLZ and CLZ had similar effects on blood mineral levels.

Drug-induced liver damage (DILI) is a significant challenge to public health. It exhibits a wide range of clinical manifestations that could progress to acute liver failure [91]. The incidence of drug-induced liver injury (DILI) is increasing due to the global rise in the use of psychotropic drugs, their daily long-term use, the prevalent comorbidities of psychiatric and metabolic illnesses, and polypharmacy in psychiatric patients [92]. Serum ALP, ALT, and AST activity levels are the most reliable laboratory biomarkers for hepatotoxicity. A previous study found that pathologically impaired hepatocytes secreted their contents, including ALP, ALT, and AST, into the extracellular space. The released enzymes finally entered the bloodstream, increasing the serum concentrations of ALT and AST in comparison to the control group [93].

In our study, we found that OLZ administration for 6 months significantly upregulated both AST and ALT levels, though they remained within the normal range; mild elevations within normal ranges do not necessarily indicate significant hepatotoxicity. Additionally, total bilirubin and direct bilirubin levels barely changed, and the albumin concentration decreased by 7%. Conversely, CLZ administration significantly upregulated ALT, AST, and ALP levels, though they remained within the normal range.

Patients suffering from psychiatric disorders often need long-term prescriptions, underscoring the need to identify the tolerated doses of and maintain adherence to those drugs [94,95]. The adverse effects of some antipsychotic medications may cause kidney injury, and their prolonged use may increase the risk of metabolic syndrome, contributing to the development of kidney diseases [94]. The diagnostic criteria for kidney injury are mostly dependent on blood urea and creatinine levels, which have been widely used for more than 60 years to estimate renal function. Both urea and creatinine are byproducts of muscle and protein metabolism, and their blood levels vary according to the health status of the kidneys [96]. In our study, we observed a notable upregulation of urea in patients treated with OLZ for 6 months; this was not observed in patients treated with CLZ. However, creatinine levels barely changed in both patient groups. Nevertheless, further investigations are required to clarify these findings.

Scientists have found that inflammation significantly contributes to the initiation and persistence of schizophrenia. In response to stressful life events and injury, cytokines are released by microglia, which are innate immune cells located in the central nervous system [97,98]. Recent studies indicate that pro-inflammatory cytokines, such as IL-1β, IL-6, and TNF-α, are elevated in the peripheral blood of patients with schizophrenia during acute psychotic exacerbations, which suggests that immunological changes may influence the clinical status following the onset of the illness [99,100]. In the present study, we found an intense increase in the levels of the inflammatory mediators IL-1β, IL-6, and TNF-α, and these elevated levels were significantly reduced by the administration of OLZ and CLZ for 6 months. Furthermore, OLZ at a dose of 5 mg had a stronger decreasing effect than CLZ at a dose of 25 mg, suggesting that the anti-inflammatory effects of both drugs play a crucial role in their therapeutic effects, in addition to their antidopaminergic effects.

## 4. Materials and Methods

### 4.1. Ethical Considerations

This study adhered to the ethical principles outlined in the most recent version of the Declaration of Helsinki by the World Medical Association in 1991 and 1996. Additionally, it followed the guidelines on good clinical practice and the relevant laws and regulations of the Ministry of Health of the Kingdom of Saudi Arabia, whichever provided greater protection for the individuals involved. The protocol, informed consent forms, and all other relevant study-related documents received approval from an independent ethics committee at the Ministry of Health of the Kingdom of Saudi Arabia (National Committee of Bioethics (NCBE); Approval No: H-04-Q-001). Participants were enrolled in the study after receiving a detailed explanation of the study protocol and providing written informed consent.

### 4.2. Study Design and Setting

This study employed a cohort prospective design. Patients admitted to the Qassim Mental Health Hospital in Saudi Arabia and diagnosed with schizophrenia in the period from July 2023 to April 2024 were checked for eligibility. Diagnosis was based on the criteria outlined in the Diagnostic and Statistical Manual of Mental Disorders, Fifth Edition (DSM-5, American Psychiatric Association, 2013). During the study, the patients did not receive specific instructions regarding diet restrictions.

The following patient demographic data were obtained: age, gender (female/male), marital status (married/unmarried), and occupational status (employed/unemployed).

### 4.3. Eligibility Criteria

#### 4.3.1. Inclusion Criteria

Each enrolled patient met the following criteria: aged between 18 and 65 years with a minimum level of positive and/or negative symptoms at the time of evaluation. The minimum threshold for positive symptoms was a total score of at least 8 on the Brief Psychiatric Rating Scale (BPRS) for the items of conceptual disorganization, hallucinations, unusual thought content, and suspiciousness (item scores on the BPRS range from 1 to 7) or a score of 4 or higher on any of these items. The minimum threshold for negative symptoms was defined as a total score of 20 or higher on the Scale for the Assessment of Negative Symptoms (SANS), where item scores range from 1 to 7.

In the present study, 150 patients were involved, and 82 met our inclusion and exclusion criteria. A total of 45 patients received CLZ according to their mental health status and the psychiatrist’s recommendation, 9 patients preferred to continue their treatment plan and follow-up in a nearby mental health hospital due to distance and transportation issues, 7 patients were administered other drugs that may have affected our outcome results (such as beta blockers and diuretics), and the psychiatrist chose to change the treatment plan during the study for 5 patients. A total of 37 patients received OLZ, 7 patients chose to continue their treatment plan in a nearby mental health hospital, 5 patients had other illnesses during the study that necessitated the administration of other drugs that may have affected the obtained results, and 1 patient was randomly excluded to ensure the same number of patients in each treatment group.

In the present study, eligible patients were randomly divided into the following study groups:

1. Healthy control group.
2. CLZ-treated group: The parameters were determined before starting clozapine treatment (25 mg) and 3 and 6 months after treatment began.
3. OLZ-treated group: The parameters were determined before starting olanzapine treatment (5 mg) and 3 and 6 months after treatment began.

#### 4.3.2. Exclusion Criteria

The exclusion criteria included a diagnosis of substance-related disorder according to DSM-5, serious suicidal risk (category 4 or higher, as determined using the Columbia–Suicide Severity Rating Scale), pregnancy or breastfeeding, a history of seizures, a history of leukopenia of unknown etiology, a leukocyte count less than 3.5 × 10^3^/μL and/or a neutrophilic granulocyte count less than 2.0 × 10^3^/μL upon entering the study, the administration of diuretics or any other drug that may have affected the examined metabolic parameters, cardiovascular disease, hepatic disease, diabetes, renal disease, current jaundice, or active hepatitis. Patients were excluded if they had previously experienced treatment failure with OLZ or CLZ due to either adverse events or a lack of effectiveness.

#### 4.3.3. Outcome Determination

##### PANSS Determination

At baseline and 3 and 6 months after CLZ or OLZ administration, the PANSS symptom severity score [101] was obtained. The total score is the cumulative sum of the scores of three subscales: a positive symptom scale (ranging from 7 to 49), a negative symptom scale (also ranging from 7 to 49), and a general psychopathology scale (ranging from 16 to 112). 

All evaluators are specialized psychiatrists with substantial expertise in assessing individuals with SCZ.

##### Laboratory Procedures

The blood concentrations of urea (Cat No#E-BC-K183-S, Elabscience, Houston, TX, USA), Creatinine (Cat No#E-BC-K188-M, Elabscience, Houston, TX, USA), ALT (Cat No# E-BC-K235-M, Elabscience, Houston, TX, USA), AST (Cat No#E-BC-K236-M, Elabscience, Houston, TX, USA), total bilirubin (Cat No# E-BC-K760-M, Elabscience, Houston, TX, USA), direct bilirubin (Cat No# E-BC-K761-M, Elabscience, Houston, TX, USA), albumin (Cat No#E-BC-K057-S, Elabscience, Houston, TX, USA), sodium (Cat No#CCRSOD1, Prestige Diagnostics, Northern Ireland, UK), potassium (Cat No#CCRPOT1, Prestige Diagnostics, Northern Ireland, UK), magnesium (Cat No# CCRMAG1, Prestige Diagnostics, Northern Ireland, UK), chloride (Cat No#CCRCHL1, Prestige Diagnostics, Northern Ireland, UK), calcium (Cat No#CCRCAZ1, Prestige Diagnostics, Northern Ireland, UK), CK-MB (Cat No# CCRCKM1, Prestige Diagnostics, Northern Ireland, UK), glycosylated hemoglobin (Cat No# E-UNEL-H0333, Elabscience, Houston, TX, USA), cholesterol (Cat No# CCRCHO1, Prestige Diagnostics, Northern Ireland, UK), HDL (Cat No# CCRHDL1, Prestige Diagnostics, Northern Ireland, UK), and LDL (Cat No# CCRLDL1, Prestige Diagnostics, Northern Ireland, UK) were determined using standard laboratory assay kits.

##### Cardiac Performance Test

Systolic and diastolic blood pressure, the electrocardiogram (ECG)-corrected QT interval (QTc), and heart rate were determined by a specialized independent cardiologist.

##### Neurotransmitter Determination

A solid-phase sandwich ELISA technique was applied to quantify the patients’ serum levels of serotonin, dopamine, leptin, and ghrelin using kits obtained from BT LAB, Shanghai Korain Biotech, Shanghai, China, with catalog numbers #E1128Hu, #EA0041Hu, #E1559Hu, and #E3091Hu, respectively.

##### Western Blot Analysis of Inflammatory Mediators

Denatured proteins were extracted from serum samples using Laemmli sample buffer with protease inhibitors (Thermo Scientific, Waltham, MA, USA), and a Coomassie protein assay kit #27813 (Sigma Aldrich, St. Louis, MO, USA) was used to determine the total protein concentration of cell lysates.

Human IL-6 Recombinant Protein (MW: 20.95 kDa; catalog #RIL6I; dilution 1:1000; Thermo Scientific, Waltham, MA, USA), Human IL-1 beta Monoclonal Antibody (dilution 1:300; eBioscience, Thermo Scientific, Waltham, MA, USA), and Human TNF-α Recombinant Protein (MW: 17.5 kDa; catalog #PHC3011; dilution 1:500; Gibco, Thermo Scientific, Waltham, MA, USA) were used to determine the IL-6, IL-1β, and TNF-α concentrations in the serum samples using the Western blot technique. A UVP transilluminator gel documentation device (Analytik Jena, Upland, CA, USA) was used to observe the membrane at 340 nm. Analytica Jena software (UVP ChemStudio, 07745 Jena, Germany) was used to determine the protein ratio, which is the ratio of the protein concentration to the total protein, for each sample.

### 4.4. Statistical Analyses

Statistical analyses were performed using the SPSS software version 22.0 (IBM Corp, Armonk, NY, USA) package for Windows and GraphPad Prism version 8 (GraphPad Software, San Diego, CA, USA). The obtained results from PANSS score, hepatic functions, renal functions, minerals concentrations, and metabolic parameters were analyzed by Mann–Whitney *U* test to obtain data scored on a two-point scale and data were expressed as the mean ± SD. Neurotransmitters and inflammatory parameters are presented as the mean ± SEM, and the data were statistically analyzed using a one-way ANOVA test, followed by Tukey’s multiple comparison test. Statistical significance was defined at *p* < 0.05.

## 5. Conclusions

The available data on the effects of OLZ and CLZ on different body systems have not been fully explored and, in some cases, are contradictory. In the present study, we compared the systemic effects of two of the most commonly used drugs—OLZ and CLZ—to facilitate their optimal use in patients with schizophrenia and different comorbidities. We found the following: Regarding the PANSS score, the drugs did not have the same clinical impacts. CLZ more strongly attenuated both general symptoms (anxiety, guilt feelings, tension, mannerisms and posturing, depression, motor retardation, disorientation, lack of judgment and insight, and disturbance of volition) and negative symptoms (blunted affect, emotional withdrawal, poor rapport, and difficulty in abstract thinking). Regarding the effects on blood minerals, we found a non-significant difference between the treated groups. The measurement of cardiac parameters showed that the OLZ-treated group had higher CK-MB levels than the CLZ-treated group. Moreover, the measurement of metabolic parameters showed that CLZ had greater effects on HbA1C, LDL, and HDL levels than OLZ. OLZ had more potent anti-inflammatory effects than CLZ, as determined by measuring IL-1β, IL-6, and TNF-α levels. A kidney function examination showed that OLZ had stronger upregulatory effects on creatinine than CLZ; as for liver enzymes, OLZ was found to have a more significant upregulatory effect on AST than CLZ. Overall, the present study obtained significant results that can aid clinicians in selecting the appropriate treatment for patients, considering their comorbidities and specific subclass of schizophrenia.

## 6. Study Limitations

Despite the usefulness of the present study, limitations persist. First, although various parameters of metabolism and schizophrenia were measured, some parameters such as those related to cardiovascular effects need to be further examined for a complete evaluation; for example, ambulatory blood pressure, troponin, continuous ECG (Holter monitoring), and heart rate variability monitoring should be conducted. Second, an extended study duration would be more valuable, and future research should assess and compare the long-term antipsychotic and metabolic effects of clozapine and olanzapine in patients with schizophrenia. Given the diversity of antipsychotic medications, further comparisons, particularly between typical and atypical antipsychotic medications, are required in future studies. When examining metabolic properties, more parameters need to be included, and the participants need to follow a restricted diet throughout the study. A subgroup analysis according to sex and age should be considered in future studies to better understand differential drug effects. Although dopamine plays a crucial role in the brain, plasma dopamine does not necessarily reflect central dopaminergic activity due to the blood–brain barrier and metabolic differences. Therefore, measuring plasma dopamine may not be a reliable indicator of brain dopamine levels or activity.

## Figures and Tables

**Table 1:** Participants’ demographic characteristics.

	Control		Olanzapine		Clozapine	
	Number	%	Number	%	Number	%
Gender						
Male	12	50	13	54.1	13	54.1
Female	12	50	11	45.8	11	45.8
Marital status						
Married	18	75	19	79.1	18	75
Unmarried	6	25	5	20.8	6	25
Employment						
Employed	17	70.8	18	75	20	83.3
Unemployed	7	29.2	6	25	4	16.6
	Mean	SD	Mean	SD	Mean	SD
Age	45.6	16.5	41.8	14.3	44.2	18.4

**Table 2:** General scale of the psychological effects in the OLZ and CLZ groups at baseline and after 3 and 6 months of treatment.

General Scale	Olanzapine Group (N = 24) (0 Months)	Clozapine Group (N = 24) (0 Months)	Olanzapine Group (N = 24) (3 Months)	Clozapine Group (N = 24) (3 Months)	Olanzapine Group (N = 24) (6 Months)	Clozapine Group (N = 24) (6 Months)
	Mean ± SD	Mean ± SD	Mean ± SD	Mean ± SD	Mean ± SD	Mean ± SD
Somatic concern	1.7 ± 0.52	2.38 * ± 0.89	1.45 ± 0.49	1.76 ± 0.69	1.3 ± 0.32	1.15 ± 0.37
Anxiety	2.2 ± 0.47	2.71 ± 0.89	1.45 ± 0.38	2.28 ± 0.48	1.6 ± 0.44	1.64 ± 0.32
Guilt feelings	1.4 ± 0.88	2.5 * ± 0.91	1.4 ± 0.28	1.78 ± 0.25	1.15 ± 0.48	1.28 ± 0.72
Tension	2 ± 0.25	2.85 ± 0.83	1.55 ± 0.24	2.5 ± 0.5	1.45 ± 0.32	1.53 ± 0.56
Mannerisms and posturing	1.75 ± 0.44	2.64 * ± 0.98	1.45 ± 0.54	1.92 ± 0.49	1.3 ± 0.40	1.28 ± 0.42
Depression	1.55 ± 0.14	2.64 * ± 0.26	1.45 ± 0.44	1.78 ± 0.25	1.45 ± 0.39	1.28 ± 0.52
Motor retardation	1.6 ± 0.27	2.64 * ± 0.56	1.45 ± 0.58	2.08 ± 0.72	1.35 ± 0.41	1.5 ± 0.71
Uncooperativeness	3.85 ± 0.48	4.85 ± 0.7	1.95 ± 0.38	2.35 ± 0.39	1.5 ± 0.40	1.42 ± 0.44
Unusual thought content	3.85 ± 0.93	4.25 ± 0.71	2.05 ± 0.35	2.35 ± 0.49	1.6 ± 0.30	1.64 ± 0.62
Disorientation	1.6 ± 0.34	2.5 * ± 0.87	1.3 ± 0.60	1.35 ± 0.43	1.05 ± 0.22	1.38 ± 0.32
Poor attention	2.05 ± 0.49	3.14 * ± 0.99	1.65 ± 0.61	2.38 ± 0.35	1.45 ± 0.40	2 * ± 0.80
Lack of judgment and insight	4.25 ± 0.46	5.07 ± 0.43	3.65 ± 0.88	3.35 ± 0.44	3.4 ± 0.64	2.71 ± 0.46
Disturbance of volition	1.95 ± 0.73	3.42 ± 0.78	1.95 ± 0.23	2.42 ± 0.73	1.4 ± 0.59	1.75 ± 0.55
Poor impulse control	3.35 ± 0.61	4.64 ± 0.86	1.75 ± 0.40	2.92 ± 0.48	1.2 ± 0.41	1.78 ± 0.38
Pre-occupation	1.95 ± 0.57	3.35 * ± 0.64	1.6 ± 0.58	2.21 ± 0.28	1.15 ± 0.36	1.46 ± 0.46
Active social avoidance	2.15 ± 0.52	3.35 ± 0.36	1.7 ± 0.62	2.85 ± 0.51	1.25 ± 0.35	1.71 ± 0.49
Total	37.2 ± 8.1	52.2 * ± 11.4	27.5 ± 7.6	35.78 ± 7.48	23.6 ± 6.43	24.92 ± 8.14
*p*-value	Baseline *p* = 0.822 value		3 months *p* = 0.098 value		6 months *p* = 0.124 value	

Analyzed using the Mann–Whitney U test. * significant difference at *p*-value < 0.05.

**Table 3:** Percentage changes in general scale scores of psychological effects between the OLZ and CLZ groups after 6 months of treatment.

Percentage Change in General Scale Scores After 6 Months	Olanzapine Group (N = 24)	Clozapine Group (N = 24)
Somatic concern	−24%	−52%
Anxiety	−27%	−39%
Guilt feelings	−18%	−49%
Tension	−28%	−46%
Mannerisms and posturing	−26%	−52%
Depression	−6%	−52%
Motor retardation	−16%	−43%
Uncooperativeness	−61%	−71%
Unusual thought content	−58%	−61%
Disorientation	−34%	−45%
Poor attention	−29%	−36%
Lack of judgment and insight	−20%	−47%
Disturbance of volition	−28%	−49%
Poor impulse control	−64%	−62%
Preoccupation	−41%	−56%
Active social avoidance	−42%	−49%
Total	−31%	−52%
*p*-value	0.187	

Analyzed using the Mann–Whitney U test. *p*-value < 0.05.

**Table 4:** The negative scale of the psychological effects in the OLZ and CLZ groups at baseline and after 3 and 6 months of treatment.

Negative Scale	Olanzapine Group (N = 24) (0 Months)	Clozapine Group (N = 24) (0 Months)	Olanzapine Group (N = 24) (3 Months)	Clozapine Group (N = 24) (3 Months)	Olanzapine Group (N = 24) (6 Months)	Clozapine Group (N = 24) (6 Months)
	Mean ± SD	Mean ± SD	Mean ± SD	Mean ± SD	Mean ± SD	Mean ± SD
Blunted affect	2.75 ± 0.9	3.35 ± 0.82	2.5 ± 0.94	3.35 * ± 0.73	2.15 ± 0.74	2.07 ± 0.58
Emotional withdrawal	2.7 ± 0.66	3.57 * ± 0.92	2.5 ± 0.94	3.42 * ± 0.98	2.3 ± 0.22	2.14 ± 0.49
Poor rapport	3.1 ± 0.95	3.78 ± 0.91	2.6 ± 0.82	3.57 * ± 0.94	2.25 ± 0.31	2.21 ± 0.31
Passive apathetic social withdrawal	3 ± 0.63	3.57 * ± 0.82	2.35 ± 0.7	3.21 * ± 0.92	2.1 ± 0.24	2.42 ± 0.63
Difficulty in abstract thinking	3.9 ± 0.49	3.85 ± 0.7	3.4 ± 0.94	3.42 ± 0.74	3.25 ± 0.26	2.71 ± 0.43
Lack of spontaneity flow of conversation	3.2 ± 0.63	3.35 ± 0.73	2.5 ± 0.74	3.21 * ± 0.8	2.1 ± 0.96	2.35 ± 0.73
Stereotyped thinking	2.5 ± 0.60	3.35 * ± 0.57	1.9 ± 0.57	3.21 ± 0.82	1.55 ± 0.49	2.21 ± 0.8
Total	20.2 ± 5.2	24.8 * ± 5.47	17.5 ± 5.65	23.4 * ± 5.39	15.7 ± 2.98	16.1 ± 3.79
*p*-value	Baseline *p* = 0.041		3 months *p* = 0.039		6 months *p* = 0.11	

Analyzed using the Mann–Whitney U test. * significant difference at *p*-value < 0.05.

**Table 5:** Percentage changes in negative scale scores of the psychological effects between the OLZ and CLZ groups after 6 months of treatment.

Percentage Changes in Negative Scale Scores After 6 Months	Olanzapine Group (N = 24)	Clozapine Group (N = 24)
Blunted affect	−22%	−38%
Emotional withdrawal	−15%	−40%
Poor rapport	−27%	−42%
Passive apathetic social withdrawal	−30%	−32%
Difficulty in abstract thinking	−17%	−30%
Lack of spontaneity flow of conversation	−34%	−30%
Stereotyped thinking	−38%	−34%
Total	−22%	−35%
*p*-value	0.129	

Analyzed using the Mann–Whitney U test. *p*-value < 0.05.

**Table 6:** The positive scale of the psychological effects in the OLZ and CLZ groups at baseline and after 3 and 6 months of treatment.

Positive Scale	Olanzapine Group (N = 24) (0 Months)	Clozapine Group (N = 24) (0 Months)	Olanzapine Group (N = 24) (3 Months)	Clozapine Group (N = 24) (3 Months)	Olanzapine Group (N = 24) (6 Months)	Clozapine Group (N = 24) (6 Months)
	Mean ± SD	Mean ± SD	Mean ± SD	Mean ± SD	Mean ± SD	Mean ± SD
Delusions	4.95 ± 1.27	5.5 ± 1.08	2.1 ± 0.78	2.92 * ± 0.54	1.55 ± 0.51	1.6 ± 0.65
Conceptual	3.6 ± 0.65	4.09 ± 0.92	1.8 ± 0.46	2.69 * ± 0.43	1.25 ± 0.44	1.5 ± 0.71
Hallucinatory	4.2 ± 0.79	4.42 ± 0.98	2 ± 0.71	3.14 * ± 0.65	1.25 ± 0.44	1.5 ± 0.67
Excitement	4.4 ± 0.61	5.14 ± 1.23	1.75 ± 0.66	2.78 ± 0.51	1.35 ± 0.74	1.28 ± 0.36
Grandiosity	2.45 ± 0.66	3.35 * ± 0.89	1.4 ± 0.65	2.36 * ± 0.46	1.1 ± 0.34	1.076 ± 0.27
Suspiciousness	4.15 ± 0.36	5 ± 1.15	2.05 ± 0.61	2.69 ± 0.97	1.65 ± 0.47	1.5 ± 0.65
Hostility	4.2 ± 0.58	5.14 ± 1.29	1.7 ± 0.70	2.57 * ± 0.54	1.5 ± 0.46	1.35 ± 0.44
Total	27.95 ± 4.92	32.64 * ± 7.54	12.8 ± 4.57	18.28 * ± 4.1	9.6 ± 3.4	9.64 ± 3.75
*p*-value	Baseline *p* = 0.424		3 months *p* = 0.325		6 months *p* = 0.542	

Analyzed using the Mann–Whitney U test. * significant difference at *p*-value < 0.05.

**Table 7:** Percentage changes in positive scale scores of the psychological effects between the OLZ and CLZ groups after 6 months of treatment.

Percentage Changes in Positive Scale Scores after 6 Months	Olanzapine Group (N = 24)	Clozapine Group (N = 24)
Delusions	−69%	−72%
Conceptual	−65%	−65%
Hallucinatory	−70%	−66%
Excitement	−69%	−75%
Grandiosity	−55%	−68%
Suspiciousness	−60%	−70%
Hostility	−64%	−74%
Total	−64%	−69%
*p*-value	*p* = 0.493	

Analyzed using the Mann–Whitney U test. *p*-value < 0.05.

**Table 8:** Total PANSS score of the psychological effects in the OLZ and CLZ groups at baseline and after 3 and 6 months of treatment.

	Olanzapine Group (N = 24) (0 Months) Mean ± SD	Clozapine Group (N = 24) (0 Months) Mean ± SD	Olanzapine Group (N = 24) (3 Months) Mean ± SD	Clozapine Group (N = 24) (3 Months) Mean ± SD	Olanzapine Group (N = 24) (6 Months) Mean ± SD	Clozapine Group (N = 24) (6 Months) Mean ± SD
Total score	85.35 ± 18.22	109.64 ± 29.73	57.8 ± 17.82	77.46 ± 16.97	48.9 ± 12.81	50.66 ± 15.68
*p*-value	0.428		0.154		0.25	

Analyzed using the Mann–Whitney U test. *p*-value < 0.05.

**Table 9:** Blood mineral levels in the OLZ and CLZ groups at baseline and after 6 months of treatment.

	Olanzapine Group (N = 24) (0 Months)	Clozapine Group (N = 24) (0 Months)	Olanzapine Group (N = 24) (6 Months)	Clozapine Group (N = 24) (6 Months)	
	Mean ± SD	Mean ± SD	Mean ± SD	Mean ± SD	*p*-Value
Blood Minerals					
Sodium level	133.9 ± 2.52	137.9 ± 3.17	131.2 ± 3.58	134.1 ± 4.75	0.647
Potassium level	3.918 ± 0.28	4.018 ± 0.35	4.042 ± 0.31	4.206 ± 0.39	0.685
Calcium level	2.251 ± 0.04	2.35 ± 0.07	2.338 ± 0.12	2.295 ± 0.11	0.774
Chloride level	100.2 ± 4.55	104.2 ± 6.28	98.06 ± 3.07	98.85 ± 2.66	0.469
Magnesium level	0.708 ± 0.04	0.808 ± 0.06	0.760 ± 0.19	0.777 ± 0.06	0.547
Blood Glucose and Lipid Profile					
HbA1C	5.80 ± 0.89	5.88 ± 1.3	6.09 ± 0.98	6.79 * ± 0.87	0.034
Cholesterol	4.2 ± 0.67	4.33 ± 1.03	4.4 ± 0.92	4.48 ± 0.78	0.685
LDL	84.05 ± 11.9	85.85 ± 21.1	87.15 ± 20.5	99.8 * ± 17.0	0.047
HDL	1.27 ± 0.24	1.29 ± 0.53	1.12 ± 0.15	1.01 ± 0.41	0.547
Kidney and Liver Function Tests					
Urea	66.38 ± 7.7	79 ± 8.67	67.42 ± 12.7	72.8 ± 14.2	0.029
Creatinine	4.11 ± 0.34	4.02 ± 0.55	4.65 ± 2	3.99 * ± 1.3	0.032
Total bilirubin	6.84 ± 1.14	7.81 ± 1.76	6.45 ± 1.09	7.7 * ± 0.9	0.041
Direct bilirubin	2.06 ± 0.56	2.65 ± 0.71	2.11 ± 1.09	2.75 ± 0.53	0.568
ALT	14.06 ± 3.05	16.21 ± 4.5	13.35 ± 3.24	15.14 * ± 4.9	0.038
AST	19.01 ± 2.05	22.35 ± 3.2	21.97 ± 2.39	27.45 * ± 3.14	0.044
Alkaline phosphatase	59.87 ± 11.4	62.57 ± 18.9	72.83 ± 19.0	76.8 ± 23.5	0.457
Albumin	43.25 ± 2.11	41.05 ± 3.76	38.93 ± 3.26	40.5 * ± 29.5	0.039

Analyzed using the Mann–Whitney U test. * significant difference at *p*-value < 0.05.

**Table 10:** Percentage changes in blood minerals relative to baseline in the OLZ and CLZ groups after 6 months of treatment.

Percentage Changes After 6 Months	Olanzapine Group (N = 24)	Clozapine Group (N = 24)	
Blood Mineral Laboratory Tests			*p*-Value
Sodium level	−2%	−3%	0.658
Potassium level	3%	5%	0.558
Calcium level	4%	−2%	0.154
Chloride level	−2%	−5%	0.214
Magnesium level	7%	−4%	0.158
Blood Glucose and Lipid Profile			
HbA1C	5%	15%	0.041 *
Cholesterol	5%	3%	0.325
LDL	4%	16%	0.032 *
HDL	−14%	−22%	0.098
Kidney and Liver Function Tests			
Urea	2%	−3%	0.151
Creatinine	14%	−1%	0.021 *
Total bilirubin	−6%	−1%	0.048 *
Direct bilirubin	2%	4%	0.214
ALT	−5%	−7%	0.096
AST	13%	24%	0.044 *
Alkaline phosphatase	22%	23%	0.521
Albumin	−10%	−1%	0.021 *

Analyzed using the Mann–Whitney U test. * significant difference at *p*-value < 0.05.

**Table 11:** Cardiac parameters in the OLZ and CLZ groups at baseline and after 3 and 6 months of treatment.

Cardiac Parameters	Olanzapine Group (N = 24) (0 Months)	Clozapine Group (N = 24) (0 Months)	Olanzapine Group (N = 24) (3 Months)	Clozapine Group (N = 24) (3 Months)	Olanzapine Group (N = 24) (6 Months)	Clozapine Group (N = 24) (6 Months)	
	Mean ± SD	Mean ± SD	Mean ± SD	Mean ± SD	Mean ± SD	Mean ± SD	*p*-Value
ECG (QTc)	404.01 ± 14.5	408.1 ± 22.4	409.4 ± 14	411.5 ± 21.6	413 ± 15.1	415 ± 23.3	0.884
SBP	125.1 ± 6.74	120.5 ± 7.19	114.8 ± 4.82	117.1 ± 5.24	123.9 ± 9.41	124.4 ± 9.65	0.745
DBP	79.21 ± 5.48	76.53 ± 6.07	73.1 ± 6.67	74.57 ± 7.64	77.35 ± 7.08	77 ± 7.48	0.652
HR	73.12 ± 7.67	75.14 ± 9.28	74.78 ± 7.89	77 ± 9.54	79.57 ± 13.2	80.23 ± 16.4	0.551
CK-MB	11.93 ± 4.88	13.03 ± 5.52	12.65 ± 4.11	13.41 ± 4.9	14.74 ± 3.47	14.8 ± 4.78	0.325

Analyzed using the Mann–Whitney U test. *p*-value < 0.05.

**Table 12:** Percentage changes in cardiac parameters between the OLZ and CLZ groups after 6 months of treatment.

Percentage Changes in Cardiac Parameters After 6 Months	Olanzapine Group (N = 24)	Clozapine Group (N = 24)
ECG	2%	2%
SBP	−1%	3%
DBP	−2%	1%
HR	9%	7%
CK-MB	24%	14%

Analyzed using the Mann–Whitney U test. *p*-value < 0.05.

**Table 13:** Effects on body weight and waist circumference in the OLZ and CLZ groups at baseline and after 3 and 6 months of treatment.

Body Weight and Waist Circumference	Olanzapine Group (N = 24) (0 Months)	Clozapine Group (N = 24) (0 Months)	Olanzapine Group (N = 24) (3 Months)	Clozapine Group (N = 24) (3 Months)	Olanzapine Group (N = 24) (6 Months)	Clozapine Group (N = 24) (6 Months)	*p*-Value
Weight (kg): mean ± SD	71.38 ± 15.1	66.03 ± 11.9	76.77 ± 23	71.42 ± 13.4	83.7 * ± 23.6	79 * ± 15.4	0.021
Waist circumference (cm): mean ± SD	97.29 ± 16.0	92.96 ± 17.9	102.2 ± 18.3	101.2 ± 17.7	108.82 * ± 18.8	106.4 * ± 17.3	0.036

Analyzed using the Mann–Whitney U test. * significant difference at *p*-value < 0.05.

**Table 14:** Percentage changes in body weight and waist circumference between the OLZ and CLZ groups after 6 months of treatment.

After 6 Months	Olanzapine Group (N = 24)	Clozapine Group (N = 24)
Weight (kg): Mean ± SD	16%	19.9%
Waist circumference (cm): Mean ± SD	11%	15%

Analyzed using the Mann–Whitney U test and chi square test. *p*-value < 0.05.

## Data Availability

The original contributions presented in this study are included in the article. Further inquiries can be directed to the corresponding authors.
